# Babesiosis at ultra-low parasitemia presenting as secondary hemophagocytic lymphohistiocytosis in a patient on B cell-depleting therapy

**DOI:** 10.1128/asmcr.00066-26

**Published:** 2026-07-24

**Authors:** Nadia Toumeh, Bismarck S. Bisono Garcia, Felicity C. Norrie, Matthew J. Thoendel, Nischal Ranganath

**Affiliations:** 1 Division of Hospital Internal Medicine, Mayo Clinic 314200, Rochester, Minnesota, USA; 2 Division of Public Health, Infectious Diseases, and Occupational Medicine, Mayo Clinic 6915, Rochester, Minnesota, USA; 3 Department of Laboratory Medicine and Pathology, Mayo Clinic 195112, Rochester, Minnesota, USA; Vanderbilt University Medical Center, Nashville, Tennessee, USA

**Keywords:** HLH, tick-borne, babesiosis

## Abstract

**Background:**

*Babesia microti*, a parasite transmitted by the *Ixodes scapularis* tick, can cause infections ranging in presentation from asymptomatic to fulminant, life-threatening disease. Ocrelizumab, an anti-CD20 B-lymphocyte monoclonal antibody used to treat multiple sclerosis, increases the risk of severe or relapsing infections with tick-borne pathogens. In rare cases, babesiosis may trigger hemophagocytic lymphohistiocytosis (HLH).

**Case Summary:**

We report a case of babesiosis manifesting in a subacute fashion with secondary HLH in a 51-year-old female on B cell-depleting therapy. She presented in December with generalized weakness and fever and was found to have pancytopenia, transaminitis, and hyperinflammatory markers consistent with HLH. A microbial cell-free DNA next-generation sequencing test detected *Babesia microti*, with subsequent confirmation by blood PCR and smear demonstrating <0.01% parasitemia. The patient improved following treatment with atovaquone and azithromycin, along with corticosteroids for HLH.

**Conclusion:**

This case highlights the heterogeneity of babesiosis and its potential to present with minimal parasitemia, yet severe systemic inflammation in immunocompromised hosts. Additionally, this case underscores the importance of maintaining a broad differential in patients on B cell-depleting agents who present with new pancytopenia and systemic inflammatory symptoms. Early utilization of microbial cell-free DNA next-generation sequencing tests may aid in timely diagnosis and treatment.

## INTRODUCTION

Babesiosis typically presents as an acute systemic illness in the spring and summer months when *Ixodes scapularis* ticks are most active. Chronic infections are uncommon and primarily occur in immunocompromised individuals. This case highlights an uncommon presentation of chronic babesiosis presenting as hemophagocytic lymphohistiocytosis (HLH) in a patient immunosuppressed with the anti-CD20 agent ocrelizumab. Workup revealed a low-level parasitemia, and her HLH improved with treatment of the babesiosis as well as corticosteroids for the HLH.

## CASE PRESENTATION

A 51-year-old female presented in December reporting several weeks of fatigue, fevers, chills, night sweats, and abdominal pain. Her medical comorbidities included relapsing-remitting multiple sclerosis on ocrelizumab, with the last dose being 3 months prior to presentation. She reported no recent travel but did report prior exposure to ticks while staying at her cabin in Wisconsin during the summer.

On presentation, the patient was febrile at 39.3°C and tachycardic. Laboratory evaluation demonstrated pancytopenia (Hgb 6.2 g/dL, WBC 1.9 × 10^9^/L with ANC 900, and Plt 75 × 10^9^/L), transaminitis (ALT 68 U/L, AST 101 U/L, and alkaline phosphatase 149 U/L), and elevated LDH (1,143 U/L) (Table 1). Peripheral blood smear demonstrated slight anisocytosis and polychromasia but no overt hemolysis or parasitemia. She met multiple HLH criteria, including fever, cytopenias, hyperferritinemia, hypertriglyceridemia, and splenomegaly, resulting in an H-score consistent with >99% probability of HLH (Table 1).

**TABLE 1:** Laboratory findings for the patient at the time of admission and 7 days after initial presentation

Laboratory values and normal values	Labs on admission	Labs 7 days after initial admission
Hemoglobin: 11.6–15.0 g/dL	6.2 g/dL	5.6 g/dL
White blood cell count: 3.4–9.6 × 10^9^/L	1.9 × 10^9^/L	1.9 × 10^9^/L
Platelets: 156–371 × 10^9^/L	75 × 10^9^/L	90 × 10^9^/L
Alanine aminotransferase: 7–45 U/L	68 U/L	50 U/L
Aspartate aminotransferase: 8–43 U/L	101 U/L	140 U/L
Alkaline phosphatase: 35–104 U/L	149 U/L	143 U/L
Lactate dehydrogenase: 122–222 U/L	1,143 U/L	1,897 U/L
Blood smear	Slight anisocytosis and polychromasia	Less than 0.01% of RBCs with *Babesia microti* infection
Ferritin: 6–175 mcg/L		4,495 mcg/L
Triglycerides: <150 mg/dL		246 mg/dL
*Babesia microti* PCR		Positive

Cross-sectional imaging noted hepatosplenomegaly with multiple hypoattenuating lesions in the spleen. Hematology was consulted, and bone marrow biopsy demonstrated abnormal myeloid configuration with no overt concern for myeloid neoplasm or evidence of hemophagocytes. PET-CT showed abnormally increased bone marrow activity without FDG-avid lymphadenopathy. She was initiated on a 7-day cefepime course (2 g every 8 hours), given concern for infectious etiology without significant improvement in symptoms.

Notable negative infectious workup included *Histoplasma* and *Blastomyces* antigen and antibodies, *Bartonella* IgG/IgM and PCR, *Coxiella* PCR, *Brucella* antibodies, EBV DNA PCR, CMV PCR, adenovirus PCR in blood, and beta-D-glucan in blood. Additionally, all blood and urine cultures were without growth. She continued to have increased weakness, and laboratory values 7 days from the initial presentation continued to demonstrate pancytopenia, with now worsening transaminitis and evidence of hemolysis and inflammation (undetectable haptoglobin, ferritin 4,616 mcg/L, and triglycerides 246 mg/dL; Table 1). Given concern for secondary HLH, she was initiated on dexamethasone with subsequent improvement in pancytopenia.

A microbial cell-free DNA next-generation sequencing (mcfDNA-NGS) test obtained on admission returned positive for *Babesia microti*, with 44,468 molecules/100 nL detected. Subsequent testing revealed positive blood PCR for *Babesia microti*, with fewer than 0.01% of red blood cells showing evidence of parasitic infection on blood smear. The patient was then initiated on atovaquone 750 mg twice daily and azithromycin 500 mg daily for 6 weeks and discharged on a prednisone taper for treatment of secondary HLH. Six weeks of treatment were elected, given the patient’s immunocompromised status; the prednisone taper was also completed over 6 weeks. At follow-up, she showed marked clinical improvement with normalization of the blood counts, and repeat *Babesia* blood PCR and smear were negative.

## DISCUSSION

Babesiosis is a protozoal infection of erythrocytes transmitted by ticks, with most U.S. cases caused by *Babesia microti*, which is transmitted by the *Ixodes scapularis* tick (1, 2). Cases are most prominent in the Northeast and upper Midwest, typically occurring between May and October, with a typical incubation period from 5 days to 4 weeks and occasionally up to 6 months (1, 3). Clinical presentation can include fever, chills, night sweats, headache, myalgia, and arthralgia. Severe manifestations of the disease include acute respiratory distress syndrome, renal failure, and disseminated intravascular coagulation (4, 5). Severe infection is more likely to occur in the elderly and immunocompromised patients, including those with advanced HIV infection, asplenia, or those receiving chemotherapy or immunomodulatory therapies (5). Immunotherapy targeting B lymphocytes, like ocrelizumab, has been associated with persistence or relapse in some babesiosis cases (4). Hematological manifestations of babesiosis are common, and some patients can develop immune system overactivation manifesting as HLH (2).

This case illustrates several atypical and clinically relevant features. First, the patient presented with ultra-low parasitemia (<0.01%) yet severe systemic inflammation. The patient’s use of ocrelizumab, a humanized monoclonal anti-CD20 antibody used for treatment of relapsing-remitting and primary progressive multiple sclerosis, likely contributed to the atypical and severe clinical disease manifestation of babesiosis (4). CD20-positive B cells are a vital component of the humoral immune response and play a critical role in clearance of parasitic infections. Impaired antibody-mediated clearance of intraerythrocytic parasites like *Babesia microti* may lead to persistent low-level parasitemia with paradoxically exaggerated immune activation. Patients with multiple sclerosis on ocrelizumab should be counseled on avoiding tick bites, and further studies are needed to guide safe reintroduction of B cell-depleting therapy following babesiosis (4).

Additionally, this case was unique in that the majority of the patient’s symptoms were due to secondary HLH rather than babesiosis, as evidenced by her clinical improvement shortly after initiation of glucocorticoids and prior to initiation of antibiotics. HLH can present in a primary or secondary form, with primary HLH being a mostly inherited deficiency of components of the immune downregulation system and more likely to be seen in children, but it can also present in adults. Secondary HLH is most commonly caused by infection, followed by malignancy, autoimmune disease, transplant, acquired immune deficiencies, and other unknown/unidentified etiologies. The diagnosis of secondary HLH remains challenging, given the lack of a specific diagnostic test to identify the disorder; a cohort of scoring systems can help predict the probability of a patient having HLH. In cases of secondary HLH, the HScore can be utilized; this scoring system includes the presence of fever, organomegaly, cytopenia, elevated triglycerides/ferritin/serum AST, decreased fibrinogen, and the presence of hemophagocytosis in the bone marrow or baseline immunosuppression (6, 7). Our patient’s HScore placed her at >99% probability of having HLH (given immunosuppression, fever, organomegaly, cytopenia, elevated ferritin/AST/triglycerides, and low fibrinogen); hence, she was treated with glucocorticoids to aid in controlling the inflammation while awaiting further diagnostics. The absence of hemophagocytes in a bone marrow aspirate is not uncommon in HLH, as these are seen in only 40% of patients at the time of disease onset (6). Identifying and treating the trigger remains the most crucial component of caring for patients with secondary HLH, since glucocorticoid therapy or immunosuppression will treat HLH symptoms but not the underlying condition (7).

Lastly, the patient’s presentation is notable in that the infection was identified using mcfDNA-NGS testing in the setting of ultra-low parasitemia, with an initial negative peripheral blood smear. The patient’s presentation in December, likely several months following a likely tick bite, contributed to diagnostic uncertainty. Plasma mcfDNA sequencing enables noninvasive detection of a broad range of pathogens and has demonstrated utility in identifying infections with low organism burden (8, 9). In this case, the rapid and unbiased nature of mcfDNA-NGS facilitated timely diagnosis and treatment, in addition to avoiding more invasive interventions, such as splenectomy in this patient. While *Babesia* PCR and serology remain a sensitive and more widely available diagnostic modality, this case highlights the additive value of mcfDNA-NGS in complex cases with atypical presentation.

This case highlights the importance of maintaining a broad differential diagnosis, including tick-borne infections, in immunocompromised patients presenting with systemic inflammatory syndrome with pancytopenia and transaminitis, regardless of seasonality. While uncommon, tick-borne infections such as babesiosis can manifest as secondary HLH. Utilizing pathogen-agnostic assays such as mcfDNA-NGS testing may facilitate timely diagnosis and management, even among those with low parasitemia levels.
